# Characterization of Cell Wall Compositions of Sodium Azide-Induced Brittle Mutant Lines in IR64 Variety and Its Potential Application

**DOI:** 10.3390/plants13233303

**Published:** 2024-11-25

**Authors:** Anuchart Sawasdee, Tsung-Han Tsai, Yi-Hsin Chang, Jeevan Kumar Shrestha, Meng-Chun Lin, Hsin-I Chiang, Chang-Sheng Wang

**Affiliations:** 1 Department of Agronomy, National Chung Hsing University, Taichung City 402202, Taiwan; anucharts@smail.nchu.edu.tw (A.S.); johntsai.nchu@gmail.com (T.-H.T.); andy.nancy0606@gmail.com (Y.-H.C.); jeevan@smail.nchu.edu.tw (J.K.S.); 2 Institute of Plant and Microbial Biology, Academia Sinica, Taipei City 115201, Taiwan; f97b42012@ntu.edu.tw; 3 Department of Animal Science, National Chung Hsing University, Taichung City 402202, Taiwan; samchiang@nchu.edu.tw

**Keywords:** *Oryza sativa*, brittle culm, biomass, cell walls, lignocellulose, mechanical strength

## Abstract

The rice brittle culm is a cell wall composition changed mutant suitable for studying mechanical strength in rice. However, a thorough investigation of brittle culm has been limited due to the lack of diverse brittle mutants on similar genetic backgrounds in cell walls. In this study, we obtained 45 various brittle mutant lines (BMLs) from the IR64 mutant pool induced by sodium azide mutagenesis using the finger-bending method and texture profile analysis. The first scoring method was established to differentiate the levels of brittleness in rice tissues. The variation of cell wall compositions of BMLs showed that the brittleness in rice primarily correlated with cellulose content supported by high correlation coefficients (*R* = −0.78) and principal component analysis (PCA = 81.7%). As demonstrated using PCA, lower correlation with brittleness, hemicellulose, lignin, and silica were identified as minor contributors to the overall balance of cell wall compositions and brittleness. The analysis of hydrolysis and feeding indexes highlighted the importance of diversities of brittleness and cell wall compositions of BMLs and their potential applications in ruminant animals and making bioenergy. These results contributed to the comprehension of brittleness and mechanical strength in rice and also extended the applications of rice straw.

## 1. Introduction

Rice is one of the main staple crops of the world, and its production can reach up to 782 million metric tons annually [1]. Rice straw is a by-product of rice production. For every 1 kg of rice grains, approximately 1 to 1.5 kg of rice straw is generated, accounting for roughly a billion metric tons of rice straw yearly [2]. Because of the limited usage and absence of economic benefits, rice straw is considered agricultural waste. Since it has low digestibility, it requires a long time to decompose, which limits multiple crop seasons in a year or influences rice growth in the next crop season. Therefore, farmers burn the rice straw in paddy fields after harvesting, but this method releases detrimental particles and causes severe air pollution [2,3]. Many countries use policies to ban rice straw burning, yet another problem appeared when the rice straw was left in the paddy field.

An alternative method for disposing of the rice straw is allowing it to degrade naturally in the paddy field [4]. However, this approach may lead to a delayed start of the rice crop season due to the slow degradability nature of the rice straw caused by its secondary cell wall compositions, including cellulose, hemicellulose, and lignin [5,6]. The slow degradability in rice originated from cellulose, which forms insoluble, crystalline microfibrils that exhibit high resistance to enzymatic hydrolysis [7]. In addition, lignin covers cellulose and hemicellulose to form a complex structure called lignocellulose, which hinders digestibility [8]. Silica content, which is also high in rice, reduces the degradability of rice straw in the rumen by preventing the colonization of microorganisms [9]. Although chemical treatment increases the rice straw’s digestibility, it is costly and may cause environmental hazard issues or livestock health [10]. Therefore, modification of cell wall compositions could potentially accelerate the degradation of the rice straw [11,12]. This may practically make the straw burning unnecessary and reduce the drawbacks of abandoned straws. Moreover, it could potentially expand its applications.

Changing cell wall compositions causes easily breakable rice mutants, including namely brittle culm (*bc*) [12], fragile plant (*fp*) [13], and fragile culm (*fc*) [14]. The breakable tissue might include culm, leaf, node, and sheath, and some of the mutants were named brittle node [15,16] and brittle sheath [17] according to the corresponding brittle tissues. Brittle culm mutants showed reduced breaking force and thickness of the sclerenchyma cell wall [18,19], such as flexible culm (*fc*) mutant [20], did not clearly show brittleness traits despite decreasing breaking force and cell wall thickness [21].

Brittle culm (*bc*) mutants have been reported to be generated by mutagens, including chemical, biological, and physical agents, as shown in Table 1. There were at least 57 mutants from 27 wild types. Despite the fact that various brittle culm mutants were previously reported, the association between brittleness, mechanical strength, cell wall compositions, and morphological traits remains unclear, as it is challenging to make a direct comparison of the results across studies due to different experimental parameters used (Table 1). The lack of understanding about brittleness and cell wall compositions in rice resulted in having fewer brittle rice varieties for farmers [22].

To better understand brittle culm mutants and their properties, a significant number of stable brittle culm lines generated from the same genetic background, which will provide a more precise comparison is required. Therefore, this study aimed to apply the diverse brittleness mutant lines from a similar genetic background and to characterize the relevance of brittleness traits using similar parameters for better comprehension. Additionally, this study explored the potential of brittleness to enhance rice production and improve rice straw disposal in the field.

## 2. Results

### 2.1. Qualitative and Quantitative Phenotyping of Brittle Mutant Lines

**Figure 1:**
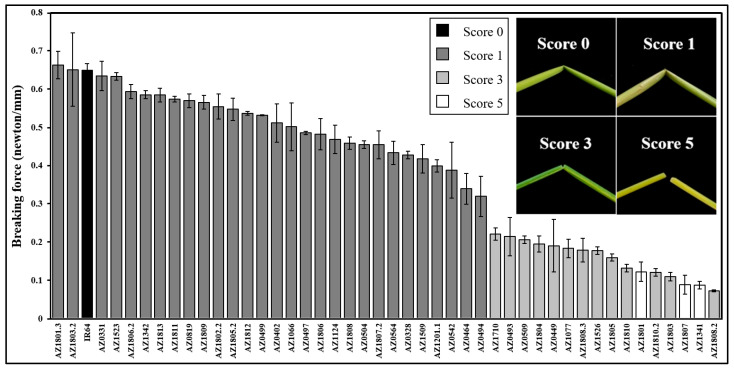
The breaking force of a fresh flag leaf of 45 BMLs and IR64 at the maturity stage. Breaking force (N/mm) was the highest force required to break the sample when using the texture profile analyzer (TPA) divided by its leaf width. The error bar is SD obtained by three repetitions (*n* = 3).

The IR64 rice mutant pool (including >1800 lines) was screened to identify mutant lines showing the brittleness trait, and 45 mutant lines with diverse brittleness levels were derived (Figure S1). The brittleness trait was stable for at least 15 crop seasons and the mutant lines were named as brittle mutant lines (BMLs) thereafter. To classify brittleness levels of the BMLs, the phenotypes after finger-bending were used. The wild type (IR64) was classified as the non-brittleness group (score 0). At the same time, thirty-one, eleven, and three BMLs were distinguished into the (score 1) low-, (score 3) moderate-, and (score 5) high-brittleness groups, respectively (Table S1). To more precisely analyze, the force required to break representing the mechanical strength of BMLs was measured using a texture profile analyzer (TPA). Using the fresh flag leaf at the maturity stage, the breaking force index of the IR64 was 0.65 ± 0.02 N/mm, while the BMLs ranged from 0.66 N/mm (102.08% of WT) to 0.07 N/mm (11.10% of WT) (Figure 1). The results indicated that the BMLs exhibit a full range of brittleness in the IR64 mutants.

**Figure 2:**
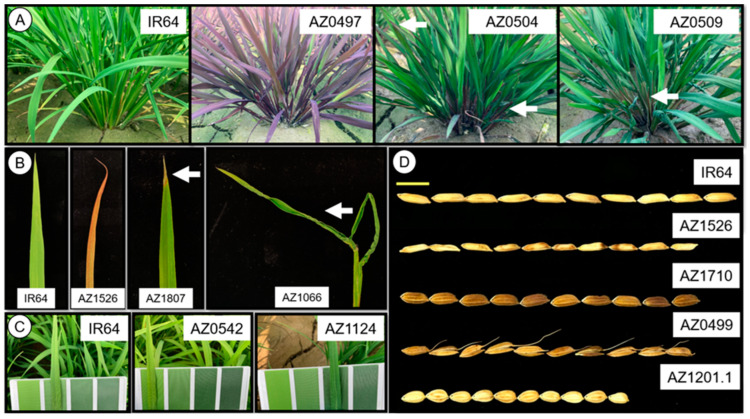
Diverse phenotypes of the BMLs from the IR64 mutant pool. (**A**) Pigmentation diversity in different tissues. (**B**) Diversity of leaf character. (**C**) Diversity of leaf green intensity by IRRI’s leaf color chart. (**D**) Diversity of grain characters (Bar = 1 cm).

The 45 BMLs also showed a variation of qualitative and quantitative morphological traits. The 54 morphological traits of BMLs and wild types were evaluated (Table S2). The qualitative traits of BMLs, including anthocyanin appearance, leaf shape, leaf greenness, grain shape, grain color, and awning, were different from the wild type (IR64) (Figure 2). When referring to the anthocyanin appearance trait, while IR64 was green, the AZ0497 displayed a uniform purple color, and both AZ0504 and AZ0509 were partially purple (Figure 2A). When examining the leaves, AZ1526 was similar to the leaf bronzing from Fe toxicity, while AZ1807 showed withering at the leaf tip, and AZ1066 displayed a twisted and curled leaf (Figure 2B). Moreover, the leaf color of some mutant lines exhibited variations of both lighter and darker shades compared to that of the IR64, such as AZ0542 and AZ1124 (Figure 2C). In addition to the traits already mentioned, the grain shape of some mutant lines was also different from the wild type. For instance, the grain of AZ1526 and AZ0499 were smaller than IR64, and AZ0499 had an awn. The grain of AZ1710 and AZ1201.1 were wider than IR64, but AZ1710 was a dark pericarp, and AZ1201.1 was a very short grain (Figure 2D). In addition to the qualitative traits, the quantitative traits of BMLs such as leaf length, culm length, and fertility were also different from IR64. Among 16 quantitative traits, the second highest coefficient of variation (CV) after the breaking force (49.20%) was found in lignin with 43.41%. The lignin content of BMLs ranged from 1.26% to 7.00% with an average of 3.40%, while the lignin of IR64 was 3.20 ± 0.73% (Table 2 and Table S2). Moreover, the dendrogram was drawn using the 54 morphological traits to demonstrate the distinction between BMLs, the wild type, and the variation among BMLs (Figure S2). The results indicated that the morphological traits and diversity of BMLs were identified.

### 2.2. Correlations Between Morphological Traits

**Figure 3:**
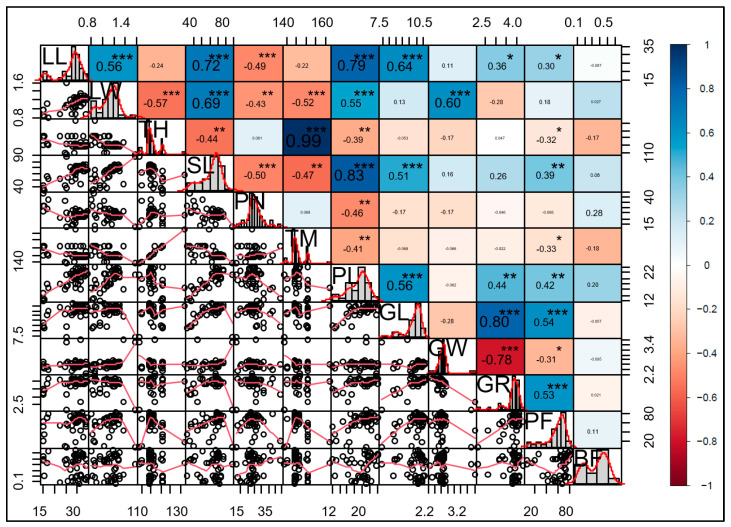
Correlation coefficients of phenotypes of BMLs. Larger numerical values and sizes indicate a stronger correlation. LL, leaf length; LW, leaf width; TH, time to heading date; SL, stem length; PN, panicle number; TM, time to maturity; PL, panicle length; GL, grain length; GW, grain width; GR, grain L/W ratio; PF, percentage of fertility; BF, breaking force. The interpretation of coefficient intervals: 0–0.19 (very low), 0.2–0.39 (low), 0.4–0.59 (middle), 0.6–0.79 (strong), and 0.8–1.0 (very strong). The asterisk indicates a significance (* = *p* < 0.05; ** = *p* < 0.01; *** = *p* < 0.001).

The association between morphological traits of the BMLs revealed that some traits were associated, while the breaking force (BF) representing brittleness weakly correlated to other traits. The BF showed a weak correlation (*R* < −0.28) with quantitative traits. There were also no qualitative traits related to the brittleness trait (Figure S3). The panicle length showed a strong and very strong positive correlation (*R* = 0.79 and 0.83) to the leaf length and stem length, respectively. Strong positive correlations were found between the grain length and leaf length (*R* = 0.64) and between the grain width and leaf width (*R* = 0.60). The results indicated that a variation in the cell size was consistent in the whole plant. The grain length showed a solid positive correlation (*R* = 0.8) to the grain length/grain width ratio (GR). In contrast, the grain width showed a strong negative correlation (*R* = −0.78) to the L/W ratio. The result indicated that the grain length played an essential role in the grain shape of the IR64 (*indica*, long grain) background rice (Figure 3).

### 2.3. Association of Brittleness, Breaking Force, and Cell Wall Compositions

**Figure 4:**
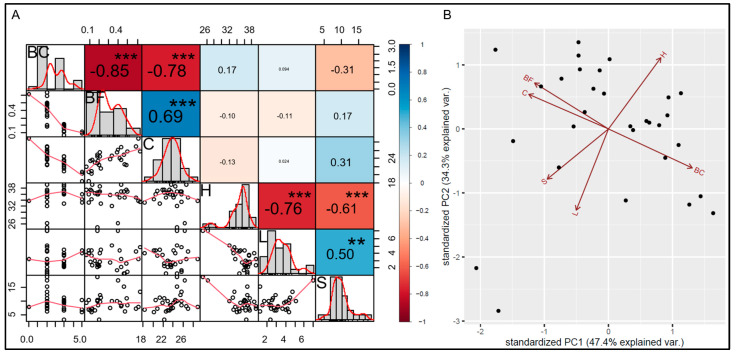
Correlation coefficient and principal component analysis of the cell wall compositions, breaking force, and brittleness score. (**A**) Correlation between the cell wall compositions: cellulose, hemicellulose, lignin, silica, brittleness score, and the breaking force of the flag leaf of brittle culm mutant lines was illustrated. Larger numerical values and sizes indicate a stronger correlation. The interpretation of coefficient intervals: 0–0.19 (very low), 0.2–0.39 (low), 0.4–0.59 (middle), 0.6–0.79 (strong), and 0.8–1.0 (very strong). The asterisk indicates a significance (** = *p* < 0.01; *** = *p* < 0.001). (**B**) Principal component analysis (PCA) biplot of BMLs on the phenotypic variables (arrows). The first two (PC1 + PC2) components accounted for 81.7% of the variance.

A significant change in cell wall compositions in the BMLs when compared to the IR64, i.e., 64–91% in cellulose, 74–116% in hemicellulose, 39–219% in lignin, and 42–239% in silica contents was observed (Table 2). To further identify the association between cell wall compositions and other parameters, including brittleness and breaking force of the representative BMLs were compared. In addition, the correlation coefficient was calculated and the PCA was performed. The correlation coefficients were calculated between cellulose, hemicellulose, lignin, silica, breaking force, and brittleness score. The brittleness score and breaking force were negatively correlated (*R* = −0.85), i.e., breaking force decreases as brittleness increases, (Figure 1 and Figure 4A). The cellulose strongly correlated significantly to brittleness score and breaking force (*R* = −0.78 and *R* = 0.69, respectively). On the other hand, hemicellulose, lignin, and silica had a very low and low correlation to brittleness score and breaking force (*R* < 0.31). However, hemicellulose and lignin showed a significantly strong negative correlation (*R* = −0.76). The hemicellulose also showed a significantly strong negative correlation to silica (*R* = −0.61). In addition, lignin showed a significant middle correlation to silica (*R* = 0.50) (Figure 4A). The PCA described that the breaking force and cellulose were the same group that was negative to brittleness score while the silica and lignin group was negative to hemicellulose, which explained 81.7% proximately (PC1 + PC2) (Figure 4B). The results indicated that the cellulose content is the major factor affecting change in mechanical strength, while the balance between hemicellulose, lignin, and silica was less associated with the brittleness of BMLs, compared to cellulose content.

### 2.4. Evaluation of the Brittle Mutant Lines for Machinery Production and Its Potential for Applications

**Figure 5:**
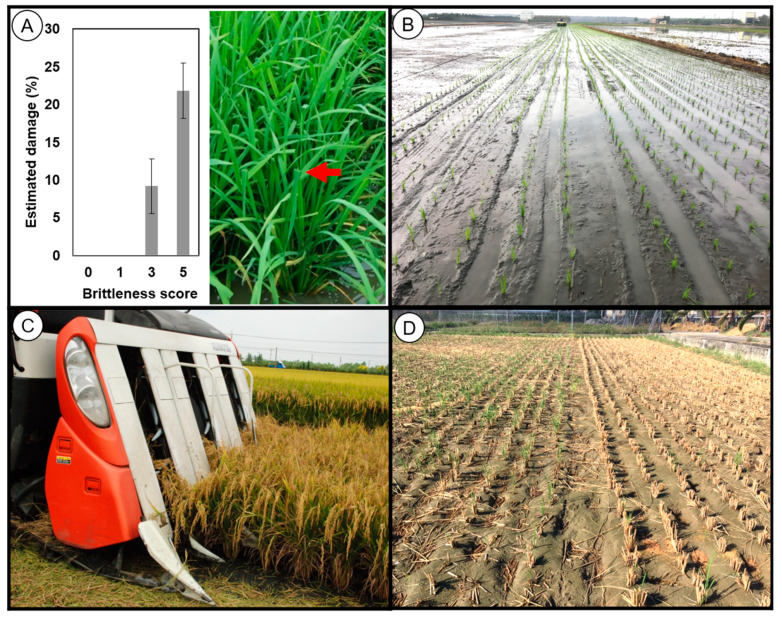
The practice of BMLs for rice farming machinery. (**A**) Bar chart showing the average percentage of leaf damage in each brittleness score group after the Megi typhoon in 2016. The red arrow pointed to the damaged leaves. (**B**) Seedling of the brittle mutant line (AZ1805) was transplanted using a transplanting machine. (**C**) The grain of the brittle mutant line was harvested using a combiner. (**D**) The degradation of stubble of the brittle mutant line (**Left**) was faster than the wild type (**Right**).

The BMLs showed the potential for rice production improvement in the paddy field. The concern with the brittle culm mutant is that it may collapse after heavy wind and rain, such as a typhoon. On 27th September 2016, super typhoon Megi, with a speed of approximately 215 km/h hit Taiwan. Therefore, the effect on BMLs was observed after the typhoon had passed. A lodging plant was not found in BMLs, indicating that the brittle rice is strong enough. Although the BMLs did not collapse, broken leaves were found. The different brittleness levels may exhibit different damage severity. Thus, the estimated damage percentage of the broken leaves was calculated. The results showed that the mutant’s leaves, which had a higher brittleness score, were more broken than those with a lower brittleness score (Figure 5A). To convince farmers that the brittle mutant could withstand rice farming types of machinery, the AZ1805 was planted by transplanting machine and harvested by the rice combiner. The results showed that the AZ1805 has no issue in rice production by machinery (Figure 5B,C). After harvesting, the brittle mutant showed faster degradation of stubble than the wild type (Figure 5D). The results showed that the BMLs have a high potential for rice production.

**Figure 6:**
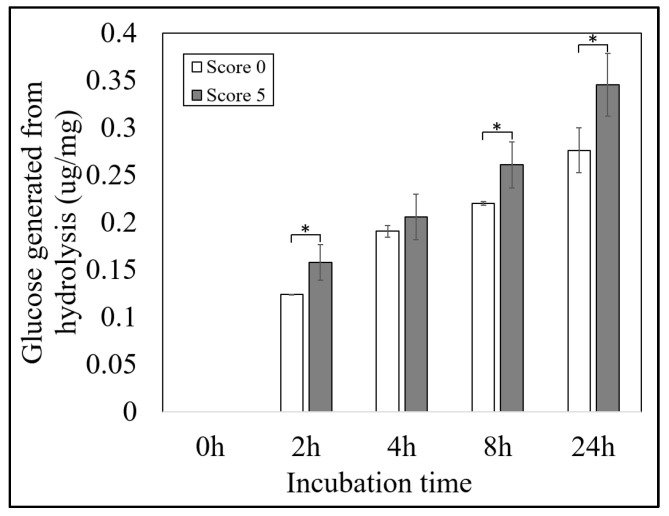
The hydrolysis means the wild type (IR64, score 0) and BMLs (score 5) within 24 h of incubation. * = significant difference by t-test (*p*-value < 0.05).

The rice straw of BMLs also showed the potential for digestion. The dry matter intake (DMI), digestion dry matter (DDM), and relative feed value (RFV) were calculated to compare the quality of forages. A direct correlation between DMI and the brittleness score was observed (Table 3). The results indicated that the BMLs rice straw with a score of 5 can be consumed in a larger quantity by ruminant animals than other scores, and those with scores of 3 and 1 can be eaten more than the score 0 (wild type). Similar to the DMI index, the DDM of score 5 was the highest followed by scores of 3, 1, and then 0. In addition, BMLs with a score of 5 had the highest DDM values, followed by scores of 3, 1, and 0. The DDM result indicated that the higher brittleness score is more digestible. The RFV showed that the higher brittleness score was more suitable than the lower score (Table 3). According to the RFV calculation results, the BMLs, with a brittleness score of 5, were chosen for hydrolysis analysis. Our results showed that the level of glucose generated from the hydrolysis of BMLs with a high brittleness score (score 5) is significantly higher than that of wild type (score 0) (Figure 6). The results showed that BMLs have the potential for digestion, which is beneficial for further applications.

## 3. Discussion

### 3.1. Screening of Diverse BMLs in the NaN_3_ Mutation Pool

The diverse BMLs generated from the IR64 variety by NaN_3_ mutagenesis increased variations of the brittleness trait and enabled the comparison among brittle mutants feasible. Germplasm diversity is an important key to success in a breeding program. Breeders commonly used mutagenesis to induce germplasm diversity [57,58,59,60]. Although various brittle culm mutants have been reported, no reports were related to brittleness and morphological trait diversity [12,30,42]. To the best of our knowledge, our study is the first to study this interesting question with many diverse BMLs derived from the same genetic background (Table 1) covering all the possible brittleness ranges that can be generated by the IR64 variety (Figure 1).

The diversity in BMLs provides insights into the differentiation of brittleness. Previously, the appearance of brittleness was used for phenotyping only by a yes/no response. Due to the diversity of BMLs, the different levels of brittleness were found and can be separated into four categories. Using breaking force per millimeter width of the flag leaf to clarify brittleness in more detail, a little gap was found between score 1 (AZ0494) and score 3 (AZ1710). Moreover, the breaking force of score 1 was relatively close to score 0, while score 3 was relatively close to score 5 (Figure 1). This indicates that the score 3 and 5 mutant lines can be considered “brittle” mutants [12], but the characteristic of the score 1 mutant line is more similar to the “flexible” mutant, which shows a decrease in breaking force [20].

The BMLs also showed a diversity of morphological traits that provided the information to convince people that using brittleness traits in rice production has no drawbacks. The variations of anthocyanin, leaf shape, leaf color, grain shape, leaf length, leaf width, culm length, panicle length, panicle number, and fertility were found in the BMLs. They showed no association with the breaking force (brittleness trait) (Table 2, Figure 2 and Figure 3). Although some characteristics appeared in many BMLs such as dwarfism in AZ0328, AZ0497, AZ0499, AZ0504, AZ1066, and AZ1124, drooping in AZ1801 and AZ1807, and low fertility in AZ1201.1 and AZ1710, and those traits can be discarded during a breeding program. Their diversity of morphological characteristics indicated that several genes not involved in the cell wall composition biosynthesis were also mutated simultaneously. Interestingly, NaN_3_ can generate various diverse brittleness and morphological traits in rice [57,58,60].

### 3.2. Development of Methodology for Brittleness Trait Investigation Using BMLs

The simple scoring method for brittleness evaluation in rice was first established using fresh flag leaf characteristics of diverse BMLs by finger bending. Unfortunately, there is a lack of investigation methods for comparison among brittle mutants due to the research limitations [32,51,55]. This research gap is possible because brittle mutants of interest should have enough mutants and share several parameters, such as similar genetic backgrounds and investigation methods. Previously, although, the brittle culm mutants (at least 12 mutants) that came from the similar Nipponbare background, such as *bc11* [19], *S1-60* [28], bc16 [29], S1-24 [18], *Bc19* [30], 5 Tos17-mutants [42], *C8* [43], and *gnt1* mutants [44], the comparisons were not conducted, as these mutants were investigated using different tissues and growth stages (Table 1). Consequently, we used a superficial structure tissue that was a flag leaf at the maturity stage of 45 diverse BMLs to develop brittleness scoring. The results from the developed brittleness score method were consistent with the breaking force by using TPA (*R* = −0.82) (Figure 4). This method was also used on other populations, and there were 23 mutant lines from the TNG67 mutant pool (*japonica*), also showing different scores of brittleness (Figure S4, Table S3), which supported the versatility of this method. However, due to human sensitivity, only non-, low-, moderate-, and high-brittleness were defined even though the brittleness of BMLs was diverse. Therefore, a new investigator can use the method practically with some training. The brittleness score method could help the breeder in the selection of brittle rice varieties.

### 3.3. Explanation of Mechanical Strength in Rice by Cell Wall Compositions of BMLs

With the advantages of the diversity of BMLs from the same wild type, the comparison of cell wall composition demonstrated that rice’s mechanical strength depends on cellulose and the combination of hemicellulose, lignin, and silica. Although the secondary cell wall in rice consists of cellulose, hemicellulose, and lignin [12,61,62], the results showed that only cellulose had a strong correlation to brittleness and a middle correlation to breaking force, similar to those reported in the literature (Figure 4A) [18,63]. Several studies claim that a decrease in cellulose content is compensated by increased hemicellulose to balance the cell wall structure. However, this claim has not yet been proven because of the lack of comparable mutants [13,23,33]. Moreover, different wall compositions of each variety make it challenging to compare mutants from different genetic backgrounds [64]. We found that not only hemicellulose but lignin and silica also changed in the BMLs to balance total compositions (NDF of BMLs = 64.93 ± 2.94) to bring the NDF (total fiber) value closer to that of wild type (NDF of IR64 = 65.84 ± 0.98) (Table S2) [64,65]. The result provides insights into the changes in hemicellulose and lignin in the mutants that decreased cellulose content [23,51]. These results indicated that a single cell wall composition is not solely responsible for the overall mechanical strength. While cellulose played the most crucial role, hemicellulose, lignin, and silica also exhibited independent effects on mechanical strength (Figure 4B) [19,54,66]. These findings benefit on evaluation and balancing of the mechanical strength and cellulose content in rice that affect further applications such as very low cellulose, but high hemicellulose rice straw may be good for livestock; however, rice plants may be too weak. For further analysis, gene identification of the BMLs will be applied to gain a better understanding of mechanical strength in rice. The results may provide knowledge to design the amount of each cell wall composition in rice straw efficiently, which expands the applications for sustainable agriculture.

### 3.4. Potential of the BMLs in Rice Production and Rice Straw Application

The diverse BMLs showed potential for rice production. Based on our knowledge, farmers are concerned that the brittle rice might be susceptible to damage from strong winds, rain, and machinery applications. Moreover, despite the fact that the brittle culm mutant was defined as lodging-resistant material because the plant height of the mutants was lower than the wild type [32,51,55], and there has been a lack of research on brittle rice resistance to environmental stress. In this study, the BMLs showed sufficient strength, as they did not collapse even after the super typhoon (Figure 5A). Even though the BMLs with the high brittleness (score 5) did not experience lodging, the damage was relatively high. On the other hand, those with low brittleness (score 1) showed the most minor damage and thus may not reflect the real potential of brittleness. Therefore, the BML with moderate brittleness (score 3) was chosen for rice production through mechanization. The result indicated that the BML was also strong enough for rice farming machinery, especially transplanting and harvesting machines (Figure 5B,C) [63]. Although the machinery is practical, the design, particularly for the brittleness variety, still requires a smoother operating machine to minimize loss and collect the rice straw immediately after harvesting for its best quality. The rice straw is primarily left in the paddy field, but the degradation process is slow [5,67]. Our results showed that the rice straw degradation of the brittle mutant was faster than the wild type (Figure 5D) [51]. Therefore, the brittle mutant rice straw can feed livestock to provide more nutrition than the non-brittleness rice straw or substitute some hay to reduce cost (Table 3, Figure 6) [68]. Moreover, the brittle mutant rice straw can also generate alcohol for bioenergy [69]. The brittle rice straw needs less effort for size reduction and low lignin rice straw may skip pre-treatment steps that use alkali or acid.

## 4. Materials and Methods

### 4.1. Plant Materials and Growth Conditions

The IR64 mutant pool was generated using sodium azide (NaN_3_) (Merck Ltd., Taipei, Taiwan) mutagenesis by treating the rice seed of the IR64 *indica* rice variety [70,71]. The mutant pool (>1800 mutant lines) obtained by at least 15 generations of self-pollination was transplanted in the experimental paddy field of National Chung Hsing University, Wufeng, Taichung City, Taiwan, to acquire mutant lines with fixed morphological traits. Single seedlings were transplanted twice a year, i.e., from February to May and August to November, respectively, as Taiwan’s first and second cropping seasons. The soil composition comprised 46.5% sand, 43.6% silt, and 9.9% clay. Fertilizers, including N (21-0-0), P_2_O_5_ (0-18-0), and K_2_O (0-0-60) (Taiwan Fertilizer Co., Ltd., Taipei, Taiwan), were applied at the rate of 125, 55, and 85 kg/ha, respectively, during the soil preparation period, as well as 15, 30, and 60 days after transplanting, following the fertilization manual of rice from Taiwan Rice Research Institute (TARI).

### 4.2. Brittle Culm Mutant Screening and Developed Brittleness Score

To obtain brittle mutant lines (BMLs), the IR64 mutant pool was investigated by three investigators using the finger-bending method and brittleness score. The brittleness score was developed to differentiate brittleness levels. The levels were divided into four scores: score 0 (no brittleness; plant did not generate a crisp sound when bent), score 1 (low brittleness; plants did not break, but produced crisp sound when bent, and it can be separated after mashing by finger), score 3 (moderate brittleness; plants were easily broken, but the broken parts were not always separated), and score 5 (high brittleness; plants were easily broken, and the broken parts were separated from each other) [72].

### 4.3. Mechanical Strength Measurements

At the maturity stage, the breaking force of fresh flag leaf from wild type and BMLs (sample size of each line was nine leaves from three plants) was measured using an iDealTA Texture analyzer (specification: 5 kg load cell, 0.01 mm distance resolution, 0.1–10 mm/s movement speed range, Horn Instruments Co., Ltd., Taoyuan, Taiwan). Both sides of the leaf were fixed at the stand and sensor probe before pulling. The maximum point of force required to break the flag leaf was divided by the flag leaf width for the breaking force index (Newton per millimeter, N/mm).

### 4.4. Morphological Traits Investigation

The traits were investigated using the international guidelines for the conduct of tests for distinctness, uniformity, and stability for rice [73], as well as for the standard evaluation system (SES) for rice [74], such as anthocyanin appearance, grain shape, and so on. Moreover, various traits were also investigated, including leaf color by the color chart developed by the International Rice Research Institute (IRRI).

### 4.5. Cell Wall Composition Analysis

To prepare the samples for cell wall composition analysis, the rice straw at the maturity stage was dried in a hot air oven at 55 °C for 72 h. Then, the dried rice straws were ground to a fine powder and filtered through a 40 mesh (approximately 400 µm) sieve. The samples (0.5 ± 0.0005 g) were then put in a filter bag (F57 Filter Bag with 25 µm pore size, ANKOM Technology, NY, USA). Cell wall compositions of the sample (*n* = 3) were analyzed using neutral detergent fiber (NDF, ISO 16472), acid detergent fiber (ADF, ISO 13906), and acid detergent lignin (ADL, ISO 13906) [64] with ANKOM Technology’s protocol (https://www.ankom.com, accessed on 30 October 2022). The hemicellulose value was calculated from the difference between NDF and ADF (Hemicellulose = NDF − ADF), while the cellulose value was obtained from the difference between ADF and ADL (Cellulose = ADF − ADL). The lignin value equals ADL (including ash). Ash content is obtained by burning in the furnace at 600 °C for 6 h [75]. To analyze silica content, ash from the sample powder was dissolved following the slightly adjusted protocol of CN1879666A [76], with the absorbance (810 nm) of the solution being measured using a spectrophotometer (U-2900, HITACHI, E HONG Instruments Co., Ltd., Taipei, Taiwan). The absorbance value was converted to silica content by the equation for the constructed standard curve (y = 136.87x + 1.1134, x is absorbance value, y is predicted silica content, *R*^2^ = 0.999) using different concentrations (20, 40, 60, 80, and 100 ppm) of silicon standard solution (ICP-MS-52W-0.1X-1, AccuStandard, UNI-ONWARD Corp. New Taipei, Taiwan).

### 4.6. Estimation of Damaged Leaf After Typhoon

On the 27th of September 2016, Taiwan experienced super typhoon Megi (up to 215 km/h speed) [77]. After the typhoon passed, the numbers of damaged (broken or bent) and the total leaves in a single plant of each line were counted (*n* = 3). The estimated damage percentage was calculated by the number of damaged leaves divided by the total leaves.

### 4.7. Rice Production Testing of Brittle Mutant Line

The AZ1805 BML seedling was transferred to a paddy field located over 40 km away from Caotun Township in Nantou County to Xizhou Township in Changhua County by using a truck. The AZ1805 was transplanted using a transplanting machine (Kubota transplanter, SPV6CMD, YUCHENG INDUSTRY CO., LTD., Qingdao, China). Rice grain was harvested at the maturity stage using the combiner (Kubota Combine Harvester, PRO488/588, Yancheng Foreign Machinery Parts Co., Ltd., Yancheng, China).

### 4.8. Relative Feed Value (RFV) Calculation

To compare the value of forages, the relative feed value (RFV) was calculated. The relative feed value (RFV) can be calculated by (DDM×DMI)/1.29. To estimate the forage amount that a livestock can consume, dry matter intake (DMI) was calculated by 120/NDF value. To evaluate digestible fiber in the forages, the ADF value that included lignin, cellulose, and silica composition determines the digestibility. Digestion dry matter (DDM) was calculated by 88.9 − (0.779 × ADF value).

### 4.9. Hydrolysis and Digestion Analysis

The enzymatic hydrolysis assay for rice tissues followed the previous literature [78], with slight modifications. In brief, 10 mg of dried leaf powder was dissolved in 1 mL of 50 mM, NaOAc, pH = 5. Then, 500 μL of 1000× diluted Cellic^®^ CTec3 (Novozymes A/S, CPH, Denmark, diluted by 50 mM, NaOAc, pH = 5) was added to the sample and incubated at 50 °C for 0, 2, 4, 8, and 24 h, respectively. The solution was centrifuged at 13,000 rpm for 10 min and the supernatant was transferred to a clean new Eppendorf tube. The glucose generated by enzymatic hydrolysis was measured by the YSI2700 Biochemistry Analyzer (YSI Inc., Yellow Springs, OH, USA). For positive control, 10 mg of Whatman #1 filter paper was used and 1 μg/mg of glucose was produced at 24 h of incubation

### 4.10. Data and Statistical Analysis

The correlation coefficient between the quantitative traits and qualitative traits was calculated using Spearman’s correlations. The correlation coefficient between quantitative traits was calculated using Pearson’s correlations in the R program (Package ‘ggpubr’ Version 0.2.5). The cluster dendrogram was obtained using “hclust” and “dist” functions in R for the unweighted pair group method with arithmetic mean (UPGMA) analysis and Spearman’s coefficients. The phylogenetic tree for the genotype was drawn by R (Package ‘ape’ Version 5.4-1). Principal components analysis (PCA) was analyzed by using “prcomp” function in the R.

## 5. Conclusions

We provided information from a significant number of BMLs derived from the NaN_3_-induced IR64 mutant pool. These findings provide fundamental insights, including the first brittleness scoring method, the relationship between brittleness and cell wall compositions in rice, and the proofing of brittle rice potential for practical rice production and applications. The brittle rice straw can be used for livestock feeding and generating bioenergy as sustainable agriculture. On the other hand, the BMLs were practical for rice production because the plant from NaN_3_ mutagenesis is a non-GMO. For further analysis, the genes responsible for the brittleness of BMLs will be identified. The results will be basic knowledge for designing cell wall compositions of rice straws to fit any purpose.

## Figures and Tables

**Table 1:** Overview of brittleness investigation methods and the analysis state of cell wall compositions of the published brittle culm mutants from various wild types and sources of mutation.

Type of Mutagen	Source of Mutation	Mutant Name	Wild Type	Investigation of Brittleness		Analysis State of Compositions					References
				Growth Stage/ Tissue	TPA ^a^	Growth Stage/ Tissue	C	H	L	S	
Chemical	NaN_3_	(45) ^b^ BMLs	IR64	M/L ^c^	+	M/L	+	+	+	+	This study
	EMS	*fp2*	E-you 532	1st IN	+	2nd IN	+	+	+	+	[13]
		*Bc6*	IR68	C, L	−	2WH/C, L	+	+	+	−	[23]
		*fld1*	Jinhui10	-	+	-	+	−	+	−	[24]
		*dbc1*	Jinhui10	-	+	-	+	−	+	−	[25]
		*dwf1*	Jinhui10	-	+	-	+	+	+	+	[26]
		*fb1*	Jinhui10	-	+	-	+	−	+	−	[27]
		*bc11*	Nipponbare	2nd IN, FL	+	2nd IN	+	+	+	−	[19]
		*S1-60*	Nipponbare	Hd/1st, 2nd IN, L	+	2nd IN	+	+	−	−	[28]
		*bc16*	Nipponbare	M/C	−	M, C	+	+	+	−	[29]
		*S1-24*	Nipponbare	Hd/1st, 2nd IN, L	+	2nd IN	+	+	−	−	[18]
		*Bc19*	Nipponbare	2WH/2nd IN, FL	+	2WH, 2nd IN, FL	+	+	+	−	[30]
		*bc25*	Nipponbare	2nd IN, FL	+	C	+	+	−	−	[31]
		*bc88*	Wuyunjing7	All	−	-	+	−	−	−	[32]
		*fc116*	Zhonghua11	M/2nd IN, FL	+	C	+	+	+	−	[14]
		*bc17*	Pingtangheinuo	Hd/FL, C	+	Hd/FL, SH, C	+	−	+	−	[33]
		*bc22*	LR005	Hd/2nd IN	+	Hd/IN	+	+	+	−	[34]
Physical	^60^Co-γ rays	*bc1*	Shuang Ke Zao	1st IN, FL	+	1st IN	+	+	+	−	[12]
		*bc7(t)*	Zhonghua11	C, L	−	LGF/C	+	+	+	−	[35]
		*bcm*	Xiushui110	C, L	−	C, L	+	−	+	−	[36]
		*lcm527-1*	527	-	+	-	+	+	+	−	[37]
		*bc-s1*	9522	-	+	-	+	+	+	−	[38]
		*bc1-wu3*	Wuyujing 3	-	+	-	+	+	−	−	[39]
		*bc16(node)*	93-11	Hd/UMN	+	Hd/UMN	+	+	+	−	[16]
	γ-rays	*bc3*	Nourin8	Hd/C	−	2WH/C	+	+	−	−	[40]
	Microwave	*bc13*	Yinhuazhan	2nd IN, FL	+	R, S	+	+	−	−	[41]
Biological	Tos17	(5) mutants	Nipponbare	2nd IN, L	−	2nd IN	+	−	−	−	[42]
		*C8*	Nipponbare	-	+	-	+	+	+	−	[43]
		*Gnt1*	Nipponbare	-	+	-	+	+	+	−	[44]
		*bc26*	ZH15	-	−	M/C, L	+	+	−	−	[45]
	T-DNA	(14) lines	Tainung67	C or L	−	-	−	−	−	−	[46]
		*bc1l4*	Zhonghua11	-	−	M/IN	+	+	+	−	[47]
Other	Nature	*bc10*	Huang Jin Qin	C, L	+	C	+	+	+	−	[48]
		*bc12*	C418	2nd IN, FL	+	2nd IN	+	+	+	−	[49]
		*nbc(t)*	93-11/IRBB21	-	+	-	+	−	+	−	[50]
		*fc17*	ShenNong265	C, L	+	M/C	+	+	+	−	[51]
		*Bc18*	II-32B//Xqz B/Dular	-	+	-	+	+	+	−	[52]
		*bs1*	Nipponbare	-	−	M/SH	+	+	−	−	[53]
	Tissue culture	*bsh1*	H3774	6W/SH	−	6W/SH	+	+	+	−	[17]
		*bc15*	Zhonghua8	2nd IN, L	+	2nd IN	+	+	+	−	[54]
Collection	T-DNA and EMS	(36) lines	Nipponbare	M/4th IN	+	M/S	+	+	+	−	[55]
-	-	*bc14*	NE17	2nd IN, L	+	2nd IN	+	+	−	−	[56]

^a^ TPA = using texture profile analysis to analyze the breaking force of the plant tissues; C = cellulose; H = hemicellulose; L = lignin; S = Silica; + means the analysis was applied; − means the analysis wasn’t applied. ^b^ Number in (-) indicated numbers of brittle culm mutants (lines) in the publications. ^c^ Hd = heading stage; M = maturity stage; 2WH = 2 week after heading; LGF = late grain filling stage; All = whole-plant growth stages; 6W = 6 week stage; 1st IN = 1st internode; 2nd IN = 2nd internode; 4th IN = 4th internode; FL = flag leaf; C = culms; L = leaves; UMN = uppermost nodes; R = roots; S = shoots; SH = sheaths; + = analyzed; - = not analyzed or no data available. For the tissue of investigation, the data referred from the original paper. Some papers counted the 1st internode from the top, while some papers counted from the bottom or even not mentioned.

**Table 2:** Variability of 16 quantitative traits of 45 BMLs derived from IR64 rice variety.

Traits	IR64 (Mean ± SD)	45 Brittle Mutant Lines			
		Min	Max	Mean	CV (%)
Leaf length (cm)	31.68 ± 3.48	14.53	34.49	27.92	19.96
Leaf width (cm)	1.30 ± 0.06	0.80	1.58	1.13	14.11
Day to heading	114 ± 0.00	109	132	115.96	3.29
Culm length (cm)	70.33 ± 3.20	32.67	88.00	62.15	20.60
Day to maturity	144 ± 0.00	139	162	146.04	2.66
Panicle length (cm)	22.01 ± 2.40	12.22	24.06	19.84	15.13
Panicle no./plant	23.00 ± 1.15	11.67	44.00	24.31	24.49
Grain length (cm)	10.23 ± 0.52	7.35	10.76	9.92	7.52
Grain width (cm)	2.67 ± 0.20	2.24	3.60	2.63	8.54
Grain length/grain width	3.83 ± 0.32	2.11	4.29	3.80	10.65
Fertility (%)	77.89 ± 4.65	6.98	82.35	60.25	31.74
Breaking force (N)	0.61 ± 0.02	0.07	0.66	0.39	49.20
Cellulose content (%)	28.85 ± 1.69	18.39	28.85	23.58	8.61
Hemicellulose content (%)	33.63 ± 1.28	24.85	39.08	35.28	9.09
Lignin content (%)	3.20 ± 0.73	1.26	7.00	3.40	43.41
Silica content (%)	7.81 ± 1.41	3.31	18.67	9.45	30.50

Min = the lowest value among BMLs, Max = the highest value among BMLs, Mean = the average of 45 BMLs, and CV = Coefficient of variation of BMLs.

**Table 3:** Mean of neutral detergent fiber and acid detergent fiber, and calculation of relative feed value of each brittleness score.

Brittleness Score	NDF	ADF	DDM	DMI	RFV
0	65.84 ± 0.98	33.72 ± 2.39	62.63 ± 1.86	1.78 ± 0.07	86.65 ± 5.75
1	65.57 ± 2.93	31.00 ± 2.71	64.75 ± 2.11	1.83 ± 0.08	92.02 ± 4.87
3	65.29 ± 2.22	28.65 ± 1.28	66.59 ± 0.99	1.84 ± 0.06	95.00 ± 4.31
5	60.51 ± 2.38	25.63 ± 1.60	68.93 ± 1.24	1.99 ± 0.08	106.14 ± 5.99
Anova	*p* = 0.0175	*p* = 0.0002	*p* = 0.0002	*p* = 0.0135	*p* = 0.0002

NDF, Neutral detergent fiber; ADF, Acid detergent fiber; DMI, Dry matter intake, DDM, Digestion dry matter; RFV, Relative feed value.

## Data Availability

Data is contained within the article or Supplementary Materials.

## Supplementary Materials

The following supporting information can be downloaded at: https://www.mdpi.com/article/10.3390/plants13233303/s1, Figure S1: The plant architecture of 45 (AZ) BMLs and IR64 (wild type); Figure S2: The dendrogram of BMLs using the 51 morphological traits; Figure S3: The frequencies of BMLs showed non-related between brittleness trait and qualitative traits; Figure S4: The plant architecture of 23 (SA) BMLs and TNG64 (wild type); Table S1: The brittleness scores of 45 (AZ) BMLs and IR64 (wild type); Table S2: Morphological traits of BMLs and its wild type; Table S3: The brittleness scores of 23 (SA) BMLs and TNG67 (wild type).
