# Supplementary material for: Characterization of Cell Wall Compositions of Sodium Azide-Induced Brittle Mutant Lines in IR64 Variety and Its Potential Application
Source: Plants (Basel). 2024 Nov 25;13(23):3303. doi: 10.3390/plants13233303 (PMC11644806; doi:10.3390/plants13233303)
Supplement: Supplementary file 1 [file plants-13-03303-s001.zip › Supplementary figures.pdf]

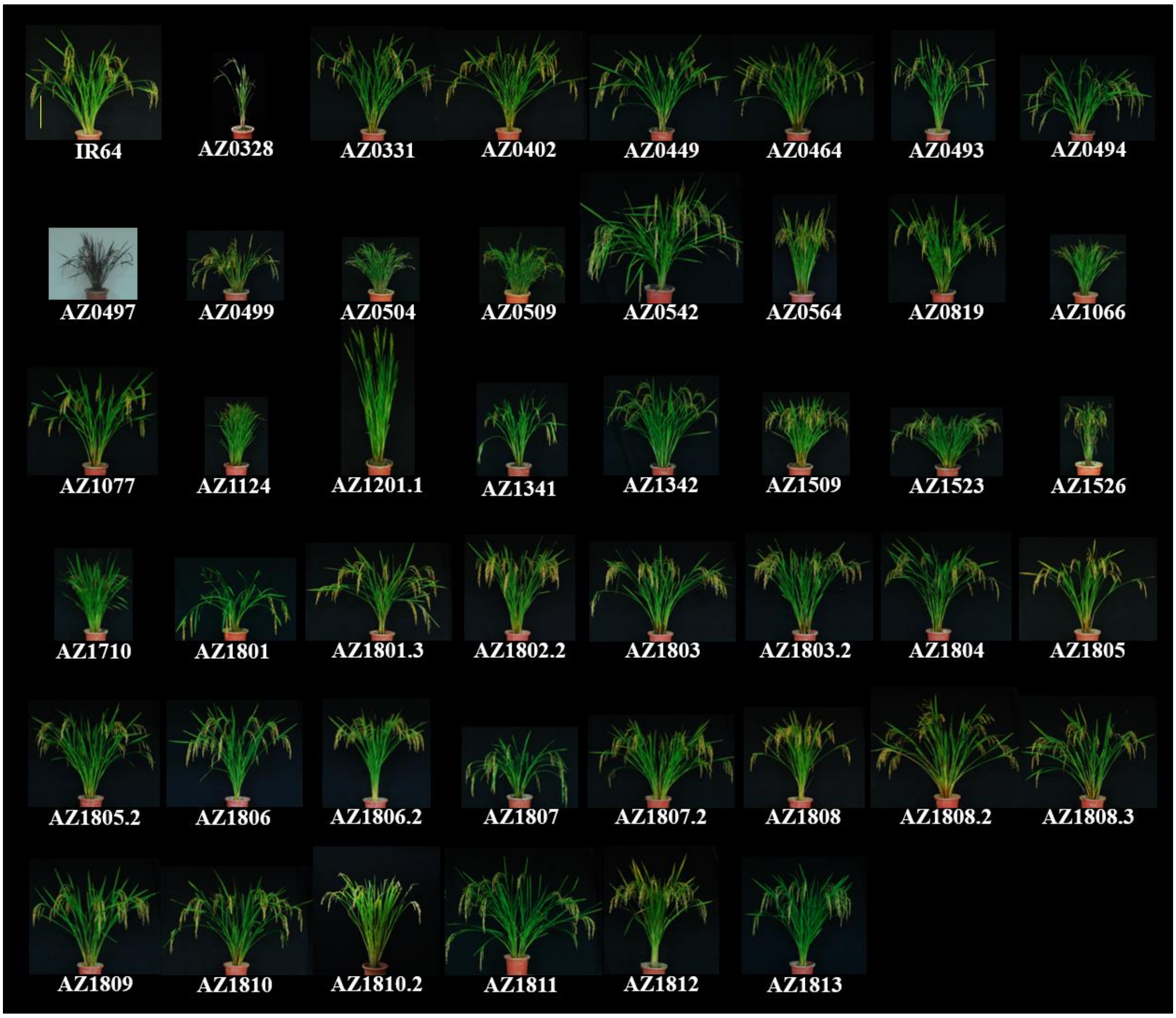

Supplementary Figure S1. The plant architecture of 45 (AZ) BMLs and IR64 (wild type). Bar = 30 cm.

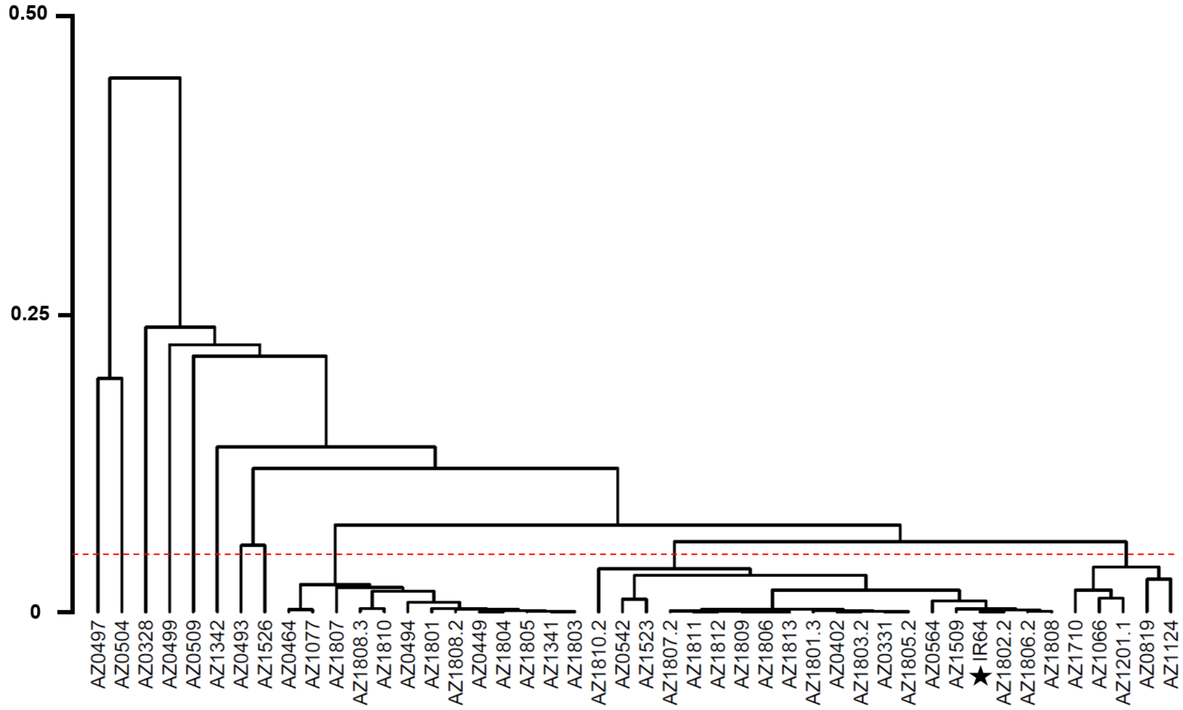

Supplementary Figure S2. The dendrogram of BMLs using the 54 morphological traits. The distance (Y-axis) was calculated using the unweighted pair group method with arithmetic mean (UPGMA) and Spearman's coefficient. The red line shows the similarity at 0.05 (95%). The asterisk shows the location of IR64 (wild type).

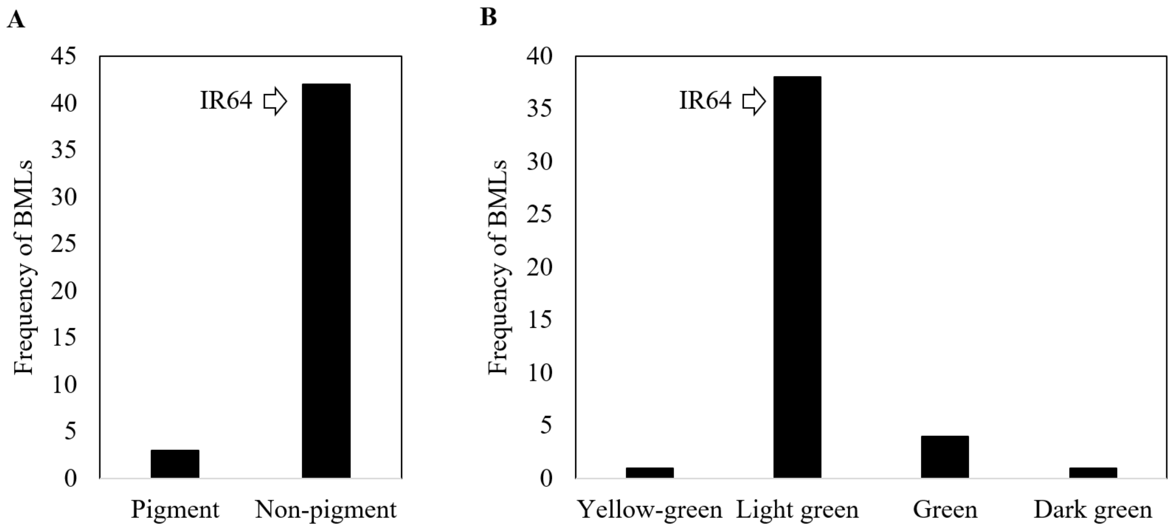

Supplementary Figure S3. The frequencies of BMLs showed non-related between brittleness trait and qualitative traits. (A) The frequency of BMLs on pigment and non-pigment. (B) The frequency of BMLs on green intensity.

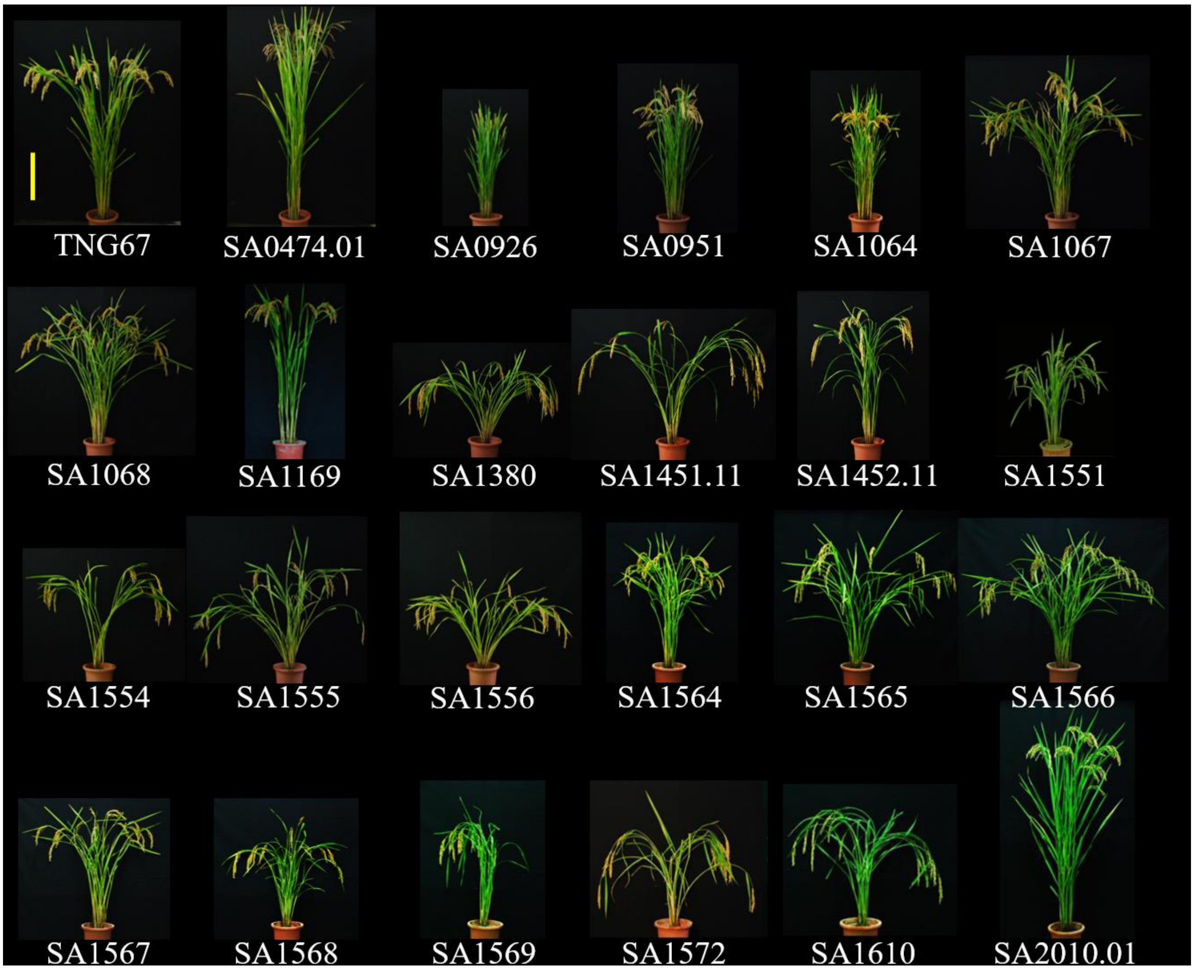

Supplementary Figure S4. The plant architecture of 23 (SA) BMLs and TNG64 (wild type). Bar = 30
