# Supplementary material for: Characterization of Cell Wall Compositions of Sodium Azide-Induced Brittle Mutant Lines in IR64 Variety and Its Potential Application
Source: Plants (Basel). 2024 Nov 25;13(23):3303. doi: 10.3390/plants13233303 (PMC11644806; doi:10.3390/plants13233303)
Supplement: Supplementary file 1 [file plants-13-03303-s001.zip › Supplementary tables.pdf]

Supplementary Table S1. The brittleness scores of 45 (AZ) BMLs and IR64 (wild type).

Brittleness score	Brittle mutant lines/ Variety
Score 0	IR64
Score 1	AZ0328, AZ0331, AZ0402, AZ0464, AZ0494, AZ0497, AZ0499, AZ0504, AZ0509, AZ0542, AZ0564, AZ0819, AZ1066, AZ1124, AZ1201.1, AZ1342, AZ1509, AZ1523, AZ1526, AZ1801.3, AZ1802.2, AZ1803.2, AZ1805.2, AZ1806, AZ1806.2, AZ1807.2, AZ1808, AZ1809, AZ1811, AZ1812, AZ1813
Score 3	AZ0449, AZ0493, AZ1077, AZ1710, AZ1803, AZ1804, AZ1805, AZ1808.2, AZ1808.3, AZ1810, AZ1810.2
Score 5	AZ1341, AZ1801, AZ1807

Supplementary Table S3. The brittleness scores of 23 (SA) BMLs and TNG67 (wild type).

Brittleness score	Brittle mutant lines/ Variety
Score 0	TNG67
Score 1	SA0474.01, SA0926, SA0951, SA1196, SA2010.01
Score 3	SA1064
Score 5	SA1067, SA1068, SA1380, SA1451.11, SA1452.11, SA1551, SA1554, SA1555, SA1556, SA1564, SA1565, SA1566, SA1567, SA1568, SA1569, SA1572, SA1610
